# Ultralong Oxford Nanopore Reads Enable the Development of a Reference-Grade Perennial Ryegrass Genome Assembly

**DOI:** 10.1093/gbe/evab159

**Published:** 2021-07-10

**Authors:** Daniel Frei, Elisabeth Veekman, Daniel Grogg, Ingrid Stoffel-Studer, Aki Morishima, Rie Shimizu-Inatsugi, Steven Yates, Kentaro K Shimizu, Jürg E Frey, Bruno Studer, Dario Copetti

**Affiliations:** 1 Agroscope, Research Group Molecular Diagnostics, Genomics and Bioinformatics, Wädenswil, Switzerland; 2 DLF Seeds S/A, Store Heddinge, Denmark; 3 Molecular Plant Breeding, Institute of Agricultural Sciences, ETH Zurich, Zurich, Switzerland; 4 Department of Evolutionary Biology and Environmental Studies, University of Zurich, Zurich, Switzerland; 5 Kihara Institute for Biological Research, Yokohama City University, Maioka, Totsuka-ward, Yokohama, Japan

**Keywords:** *Lolium perenne*, forage grasses, perennial ryegrass, genomics, genome assembly, Oxford Nanopore

## Abstract

Despite the progress made in DNA sequencing over the last decade, reconstructing telomere-to-telomere genome assemblies of large and repeat-rich eukaryotic genomes is still difficult. More accurate basecalls or longer reads could address this issue, but no current sequencing platform can provide both simultaneously. Perennial ryegrass (*Lolium perenne* L.) is an example of an important species for which the lack of a reference genome assembly hindered a swift adoption of genomics-based methods into breeding programs. To fill this gap, we optimized the Oxford Nanopore Technologies’ sequencing protocol, obtaining sequencing reads with an N50 of 62 kb—a very high value for a plant sample. The assembly of such reads produced a highly complete (2.3 of 2.7 Gb), correct (QV 45), and contiguous (contig N50 and N90 11.74 and 3.34 Mb, respectively) genome assembly. We show how read length was key in determining the assembly contiguity. Sequence annotation revealed the dominance of transposable elements and repeated sequences (81.6% of the assembly) and identified 38,868 protein coding genes. Almost 90% of the bases could be anchored to seven pseudomolecules, providing the first high-quality haploid reference assembly for perennial ryegrass. This protocol will enable producing longer Oxford Nanopore Technology reads for more plant samples and ushering forage grasses into modern genomics-assisted breeding programs.

## Introduction

Significance

Sequencing eukaryotic genomes with long-read sequencing platforms is allowing to obtain genome assemblies of unprecedented quality also for many non-model organisms. However, especially in genomes with a high amount of long repeats, completeness and contiguity are limited by the quality (accuracy and/or length) of the input data. Here we present an innovative protocol for Oxford Nanopore Technologies’ genomic plant DNA library preparation that considerably increases read length. We show how these exceptionally longer reads were key in obtaining a perennial ryegrass genome assembly with unprecedented statistics, both within its genus and among other plants of similar complexity. This work makes available a highly complete and contiguous genome assembly and the laboratory protocol necessary to produce long read data.

Over the last three years, the sequencing performance of the Oxford Nanopore Technologies (ONT) platform has dramatically increased in terms of sequencing yield, accuracy, and read length (Shafin et al. 2020; https://bit.ly/3peI7xH, last accessed May 2021). Human or bacterial substrates can nowadays be sequenced at read length N50 (the size of the shortest read that sums up to 50% of the total bases) that reaches or goes beyond 100 kb (https://bit.ly/2Ydjiq4, last accessed May 2021). As plant DNA is typically more difficult to purify and preserve at high quality, plant sequencing performance metrics lag compared with more accessible substrates. The current longest published plant ONT data (Lang et al. 2020; Zhou et al. 2020) has read N50 values around 30 kb. Though this length surpasses most of the largest repeated sequences (especially retroelements and centromeric repeats), they are still too short to resolve arrays of such sequences.

Alone or in mixture with legumes, *Lolium* and *Festuca* spp. are the main crops used as a feed source for livestock. Perennial ryegrass (*Lolium perenne* L.) is the most cultivated grass species in Western European grasslands (Wilkins and Humphreys 2003). It is a diploid (2*n* = 2*x* = 14) species with a haploid genome size of about 2.6 Gb (Kopecký et al. 2010). Like other forage grasses, perennial ryegrass is an outcrosser, meaning it retains high levels of heterozygosity. Heterozygosity and a high content in repetitive sequences are the main constrains that still hamper the development of a high-quality assembly for a forage grass (Byrne et al. 2015; Honig et al. 2016; Knorst et al. 2019). On the other hand, when striving to assemble heterozygous genomes, the two haplotypes assemble separately in allelic sequences, resulting in a diploid assembly (Copetti et al. 2021). Though it represents the true content of the nucleus of a heterozygous organism, as is, such assembly is unsuitable to be used as a haploid reference for variant calling. The closest species to the *Festuca* and *Lolium* species complex with a chromosome-scale assembly is orchardgrass (*Dactylis glomerata* L. [Huang et al. 2020]), but its incomplete nature (1.84 out of ∼2.6 Gb) and distant taxonomical placement (subtribe Dactylidinae) make it unsuitable to be used as a reference. The most proximal highly complete assemblies are *Brachypodium distachyon* and barley (*Hordeum vulgare* L.), with which perennial ryegrass shared the last common ancestor about 30 Ma (Wu and Ge 2012). The availability of a haploid or homozygous perennial ryegrass genotype would simplify the assembly of a reference genome. Haploid or doubled haploid individuals can serve such task and represent invaluable material for the development of customized breeding populations.

To compensate for the absence of a high-quality reference assembly in forage grasses, we sequenced Kyuss, a doubled haploid *L. perenne* genotype. The genotype derived from in vitro anther culture (Begheyn et al. 2017). The application of an optimized DNA extraction (Russo et al. 2021) and the development an improved sample handling protocol allowed to preserve DNA integrity, resulting in unprecedented read lengths for a plant sample. De novo assembly of the sequence data resulted in a highly complete, contiguous, and accurate genome assembly. This data set can serve as a pivotal reference assembly for genome-based studies in *Lolium* and *Festuca* biology and breeding.

## Results and Discussion

To achieve high contiguity and completeness of the Kyuss genome assembly, we optimized the standard ONT library preparation protocol. Here we report the most consequential modifications, whereas the complete protocol used in this work is available as Supplementary Material online. The salient original optimizations are the following: Reducing DNA mechanical shock during library preparation; allowing for longer elution times; and flushing the flow cell and reloading a second aliquot of library. We noticed that mixing the components by tapping and not flicking the tube helped preserving the large molecules, likely by avoiding DNA shearing and the accumulation of shorter fragments. Extending the elution time from the AMPure beads resulted in a maximization of DNA recovery, particularly preserving the high molecular weight fraction. Flushing the flow cell with Wash Solution countered pore clogging, restoring its productivity close to the initial levels and allowing to sequence more substrates on the same device. Also, we experienced that allowing for a 30-min incubation time before starting the run resulted in more active sequencing pores.

Upon base calling of the raw data, the baseline data set for all the downstream analyses consisted of 69.6 Gb of ONT data in 2,061,375 reads, having a mean read length and N50 of 33.7 and 62.6 kb, respectively. The mean base call quality was QV 10.3. The prevalence of long reads was even more clear by considering other metrics: only 6.4% of the reads are longer than 100 kb but contain about 25% of the total bases. Inspecting the current literature, such metrics are unprecedented for a plant sample.

**Fig. 1.**
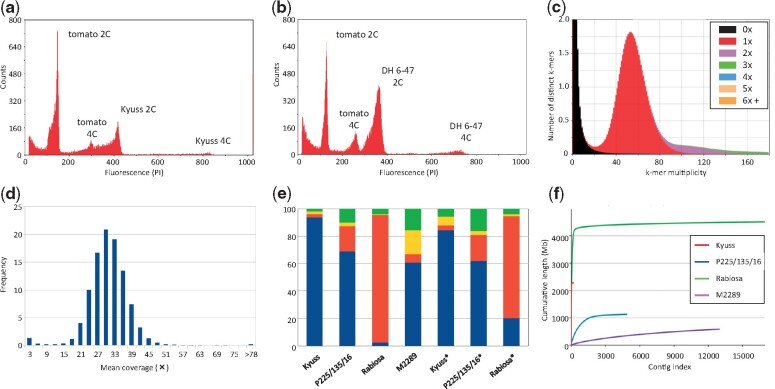
Features of the Kyuss genome and assembly. (*a*) Flow cytometry trace of Kyuss nuclei showing the occurrence of peaks at the same position as in the diploid parent (*b*) when compared with the tomato external standard. (*c*) KAT comp spectra describing the occurrence of mostly single-copy k-mers (red area) in the assembly under the main peak. The assembly is overall very complete (very small 0× area at multiplicities higher than 20) and the repeated sequences are correctly represented (purple and green enrichment values at multiples of the main peak). (*d*) Long-read coverage distribution upon read alignment. The lack of shoulders or additional peaks results entails the lack of collapsed or allelic regions in the assembly. (*e*) BUSCO analysis of the Kyuss and other public ryegrass assemblies (Byrne et al. 2015; Copetti et al. 2021; Knorst et al. 2019). Kyuss assembly shows the highest completeness in terms of conserved single-copy orthologs (SCOs), with only 4% of the models being fragmented or missing. The “Rabiosa” assembly is a diploid assembly, thus most of the SCOs are expected to be identified twice. The columns with the asterisk denote BUSCO scores for the predicted gene models. Blue: single copy, orange: duplicated, yellow: fragmented, green: missing models. (*f*) Total size and contiguity of the ryegrass assemblies evaluated by cumulative sequence length. The expected total size of the assemblies is around 2,500 − 2,700 Mb, except for Rabiosa where the diploid assembly should result in approximately 5,200 Mb. The high contiguity of the Kyuss assembly is denoted by the sharp vertical raise of the contig index approaching rapidly the total assembly size. In comparison, the “P226/135/16” and “M2289” assemblies show dramatically lower completeness and contiguity.

The size of the haploid Kyuss genome was estimated by flow cytometry and by counting k-mers from short -read data. The in silico estimation returned a value of 2.467 Gb, whereas flow cytometry estimated it at 2.720 Gb. For the sake of consistency with previous measurements in plants, we adopt the flow cytometry value as the estimated genome size of Kyuss. Furthermore, given that when compared with the tomato control the flow cytometry peak profile was the same as its parent DH 6-47 (a diploid genotype, Begheyn et al. 2017; fig. 1*a* and *b*), we concluded that Kyuss is a doubled haploid plant.

The de novo assembly of the long-read data resulted in 1,920 contigs spanning 2.28 Gb, with N50 and N90 values of 11.74 and 3.34 Mb (the 60 and 201 longest sequences), respectively. Upon correction of one chimeric contig and short-read polishing, the continuity metrics decreased slightly to 11.28 and 3.32 Mb in 1,935 sequences (table 1). The base-level accuracy was estimated to be QV 45. By intersecting k-mer abundance in the raw reads and in the assembly, the completeness was estimated at 99.39% (fig. 1*c*). Upon realignment of the long reads to the assembly, the unimodal coverage distribution entailed absence of collapsed genomic regions in the assembly (fig. 1*d*). The alignment rate was 99.89% and 89.89% for the long and short reads, respectively. Similarly, long and short reads mapped uniquely to 99.71% and 89.36% of the assembly bases, respectively. The overall lower mapping rate and breadth of coverage observed with the short read data can be explained by ambiguous alignments due to repeats and to a sequencing bias against extreme GC content regions seen with the Illumina platform (Browne et al. 2020). The assembly spanned 83.9% of the estimated haploid genome size and contained the vast majority of the single-copy orthologs expected to be present (93.5% in single copy, 2.5% in more than one copy), leaving only 2% of the models fragmented and 2% missing (fig. 1*e*). When compared with the publicly available ryegrass assemblies, Kyuss showed much higher completeness and contiguity than *L. perenne* “P226/135/16” (Byrne et al. 2015) and the *L. multiflorum* “M2289” (Knorst et al. 2019) assemblies (fig. 1*f*; table 1). Although the Italian ryegrass “Rabiosa” assembly also reached scaffold N50 values in the multi-Mb range (N50 2.9 Mb; Copetti et al. 2021), N50 is still ∼4 times shorter than Kyuss. Furthermore, Rabiosa’s diploid nature makes it not suitable to be used as a haploid reference for mapping-based analyses (e.g., SNP and SV calling, phasing). In conclusion, the Kyuss assembly is a highly complete, accurate, and contiguous representation of a haploid genome.

**Table 1:** Statistics of the *Lolium perenne* Kyuss Genome Assembly and Comparison with Other Public Ryegrass Assemblies

	Kyuss^a^	P226/135/16	Rabiosa^a^	M2289
	*Lolium perenne*	*Lolium perenne*	*Lolium multiflorum*	*Lolium multiflorum*
Reference	This study	Byrne et al. (2015)	Copetti et al. (2021)	Knorst et al. (2019)
Est. genome size (Gb)	2.720	2.068	2.464	2.500
Assembly size (Gb)	2.281	1.128	4.531	0.585
% of genome assembled	83.9	54.6	183.9	23.4
# of sequences	1,935	48,415	226,949	129,579
N50 (kb)	11,276	70	2,941	5
N90 (kb)	3,320	14	283	2
L50 (#)	65	4,908	443	37,162
L90 (#)	209	16,951	1,984	103,446

Note.—The fraction of the assembled genome is based upon the genome size estimation provided in the respective studies. In the Rabiosa assembly, most of the allelic regions are represented as separate sequences, thus reaching the diploid genome size.

a Statistics of the contigs or scaffolds before being placed on pseudomolecules.

To estimate the impact of read length on assembly completeness and contiguity, we created sets of shorter input reads by cutting the sequence at different positions, thus maintaining the same number of input bases. Though with different input read lengths the total assembly size did not change, when the reads were shorter, all contiguity values decreased (supplementary fig. 1 and table 1, Supplementary Material online). Notably, the effect was more pronounced when introducing a second cut (C20 + 20 data set, contig N50 1.9 and N90 0.5 Mb) rather than when the cut was single and more internal in the read (C30, C40, C60 data sets, contig N50 of 6.6 and N90 1.8 Mb). This can be consequence of the fact that in the C20 + 20 set more reads were cut to a shorter size, whereas in the other data sets less reads were long enough to be cut further inwards. These findings confirm the primary importance of read length in obtaining larger contigs by providing connectivity between adjacent genomic regions.

Though sequencing large and complex genomes is becoming easier and less expensive, optimizing the experimental conditions can help an optimal resource allocation. To estimate the coverage sufficient to return the best assembly possible given the current read length, assembly algorithm, and given the complexity and composition of the Kyuss genome, we assembled subsets of reads and inferred N50 values at higher coverages. Though total assembly size was not affected by the different coverage levels (supplementary table 2, Supplementary Material online), with less input data (with same average read length), contig Nx values decreased rapidly (supplementary fig. 2*a*, Supplementary Material online). We estimated that, at 52× and 70× coverage the contig N50 value has already reached 80% and 90%, respectively, of the maximum value (22.7 Mb) it could theoretically reach (supplementary fig. 2*b*, Supplementary Material online). These deducted N50 values allowed to estimate a sequencing depth threshold where the increase in contiguity is minimal, making sequencing additional bases less cost effective.

By exploiting genetic linkage of a *L. perenne* segregating population and collinearity with barley, we anchored 2 Gb (or 88% of the bases) of the assembly to seven chromosome pseudomolecules, corresponding to the haploid perennial ryegrass karyotype. Of the 235 contigs that constituted the pseudomolecules, 219 (85.5% of the bases) were oriented. Only 1,700 sequences spanning in total 274 Mb of sequence could not be anchored (N50 3.1 Mb, N90 83 kb). Besides such contigs being shorter, the unassignment to a pseudomolecule can be explained by the lack of Kyuss-specific markers (the segregating population was developed from parents that are not related to Kyuss) and by the occurrence of ryegrass-specific sequences with no counterpart in barley.

By similarity search, we identified that 60.0% of the assembly bases were composed of transposable elements and that 21.6% were constituted of uncharacterized repeated sequences. The gene annotation produced 38,868 protein-coding gene models, whose coding regions spanned 2% of the assembly. Completeness of the gene annotation set was estimated with BUSCO at 87.6% (fig. 1*e*; Seppey et al. 2019) and 92.19% with the PLAZA core gene families (411 out of 7,076 gene families missing). Both values are on par with another perennial ryegrass gene annotation (Van Bel et al. 2012; Blanco‐Pastor et al. 2020).

Here we present a method that yields exceptionally long ONT reads for a plant sample and show how read length is key in obtaining highly contiguous and complete assemblies of repeat-rich plant genomes. The resulting assembly is the first reference-grade genome map for perennial ryegrass. Its availability will enable genomics-assisted development of breeding programs.

## Materials and Methods

For complete details, see Supplementary Material online. Anther culture of the *L. perenne* genotype DH 6-47 (Begheyn et al. 2017) was used to establish doubled haploid individuals. One genotype (Kyuss) showing only one allele at three loci was selected for sequencing and clonally propagated. Ploidy level and genome size were measured via flow cytometry using tomato (*Solanum lycopersicum*) as a standard. The genome size was also estimated in silico, using short read data and Jellyfish (v2.4.2; Marçais and Kingsford 2011).

High molecular weight DNA was extracted from leaves according to Russo et al. (2021) and the ONT sequencing library was prepared with an in-house optimized Genomic DNA by Ligation (SQK-LSK109, version GDE_9063_v109_revU_14Aug2019) protocol (supplementary protocol, Supplementary Material online). The library was loaded into FLO-PRO002 flow cells and sequenced on a PromethION instrument (Oxford Nanopore Technologies, Oxford OX4 4DQ, United Kingdom). About 200 ng genomic DNA were sheared to a mean fragment size of 500 bp and an Illumina TruSeq library was produced. The library was sequenced on a Novaseq 6000 (Illumina Inc, CA) instrument in 2 × 150 bp mode.

The ONT raw data were basecalled with Guppy (v4.0.14, https://community.nanoporetech.com, last accessed May 2021), keeping reads having a minimum q-score of 7. After adapter removal, sequences shorter than 2 kb were removed. The genome was assembled with Flye (v2.7.1-b1590; Kolmogorov et al. 2019). An auxiliary assembly was generated with Shasta (v.0.6.0; Shafin et al. 2020). The Flye assembly contigs were at first polished with the built-in Flye polishing module (twice), then twice with medaka (v1.1.1, https://nanoporetech.github.io/medaka/, last accessed May 2021), and lastly by two rounds of Pilon (v1.23; Walker et al. 2014). The haploid status of the assembly was confirmed by aligning long and short reads and plotting the coverage distribution. Sequence accuracy was measured from the short-read alignment file, parsed with the SAMtools stats subcommand. The assembly completeness was assessed with KAT (v2.4.2; Mapleson et al. 2017). The completeness of gene space was assessed with BUSCO (v3.0; Seppey et al. 2019). Contigs belonging to organelles were identified by aligning the assembly to *Lolium* chloroplast and mitochondrial deposited genomes. Such contigs were kept in the assembly and the sequences were flagged. Endosymbiont sequences were identified by BlastN (BlasT+ suite, v2.9.0+; Camacho et al. 2009) against a collection of *Epichloe* and *Neotyphodium* sequences retrieved from NCBI number.

Chromosome pseudomolecules were generated with ALLMAPS (v0.7.7; Tang et al. 2015) and a genetic linkage map (Pfeifer et al. 2013) exploiting the collinearity with barley as input evidence. The *L. perenne* ESTs associated with the genetic markers were aligned to the Kyuss assembly with TBlastX. Collinearity with barley (Mascher et al. 2017) was established aligning a draft Kyuss gene annotation obtained by projecting *L. multiflorum* “Rabiosa” gene models (Copetti et al. 2021) to the Kyuss contigs. Upon aligning the two proteomes, the collinear blocks were determined with MCXScanX (v2; Wang et al. 2012). Chimeric contigs (containing stretches of more than four collinear genes aligning to a second barley pseudomolecule) were split in two or more sequences.

Repeats and transposable element were identified with RepeatMasker (v4.0.6; Smit et al.) and a custom-built *Lolium* repeat library (Copetti et al. 2021). Protein-coding genes were annotated by combining ab initio and homology-based evidence. The latter set was constituted of proteomes of annotations of closely related species (*Brachypodium*, barley, bread wheat [*Triticum aestivum* L.], perennial and Italian ryegrass) and transcripts reconstructed from publicly available RNA-Seq data from NCBI SRA. Gene predictions with homology to transposable elements were removed prior to renaming the models.

## Supplementary Material

Supplementary data are available at *Genome Biology and Evolution* online.

## Supplementary Material

evab159_Supplementary_Data
