# Supplementary material for: Ultralong Oxford Nanopore Reads Enable the Development of a Reference-Grade Perennial Ryegrass Genome Assembly
Source: Genome Biol Evol. 2021 Jul 10;13(8):evab159. doi: 10.1093/gbe/evab159 (PMC8358221; doi:10.1093/gbe/evab159)
Supplement: evab159_Supplementary_Data [file evab159_supplementary_data.zip › Supplementary_Tables_and_Figures_210504.docx]

|  | **Uncut** | **C20** | **C20+20** | **C30** | **C40** | **C60** |
| --- | --- | --- | --- | --- | --- | --- |
| **Read statistics** |  |  |  |  |  |  |
| Mean length (kb) | 33.8 | 22.5 | 17.0 | 24.4 | 26.0 | 28.5 |
| N50 (kb) | 62.6 | 22.4 | 20.0 | 30.0 | 40.0 | 51.9 |
| **Assembly statistics** |  |  |  |  |  |  |
| Total size (Mb) | 2,289 | 2,269 | 2,228 | 2,260 | 2,259 | 2,256 |
| Contig # | 2,396 | 2,304 | 3,798 | 2,011 | 2,001 | 1,996 |
| N50 (Mb) | 11.7 | 8.0 | 1.9 | 6.6 | 6.6 | 6.6 |
| N60 (Mb) | 9.6 | 6.9 | 1.5 | 5.3 | 5.3 | 5.4 |
| N70 (Mb) | 7.3 | 5.2 | 1.2 | 4.0 | 4.1 | 4.5 |
| N80 (Mb) | 5.2 | 3.6 | 0.9 | 3.2 | 3.0 | 3.3 |
| N90 (Mb) | 3.3 | 2.3 | 0.5 | 1.8 | 1.8 | 1.9 |
| L50 (#) | 30 | 87 | 361 | 103 | 103 | 101 |
| L60 (#) | 82 | 117 | 493 | 141 | 141 | 139 |
| L70 (#) | 108 | 155 | 660 | 189 | 189 | 184 |
| L80 (#) | 146 | 207 | 879 | 252 | 253 | 243 |
| L90 (#) | 202 | 283 | 1202 | 344 | 349 | 331 |

**Supplemental Table 1. Effect of read length on assembly contiguity.** To simulate a shorter read dataset, the same reads that were used for the assembly were cut at different values from the 5’ end. To reduce further connectivity power of a read, in set C20+20 the reads that were still longer than 20 kb were cut a second time at the same value. While there is no change in total assembly span, contig size (Nx values) is the metric that is primarily affected, proportionally to read length.

|  | **All reads** | **24x** | **20x** | **17x** | **14x** | **10x** |
| --- | --- | --- | --- | --- | --- | --- |
| **Read statistics** |  |  |  |  |  |  |
| # of reads (M) | 2.1 | 1.8 | 1.5 | 1.3 | 1.0 | 0.7 |
| Genome equivalents (×) | 27.8 | 24.0 | 20.0 | 17.2 | 14.0 | 9.9 |
| **Assembly statistics** |  |  |  |  |  |  |
| Total size (Mb) | 2,281 | 2,277 | 2,274 | 2,269 | 2,273 | 2,226 |
| Contig # | 2,396 | 2,311 | 2,348 | 2,559 | 2,483 | 4,084 |
| N50 (Mb) | 11.7 | 10.7 | 6.6 | 6.2 | 4.4 | 1.4 |
| N60 (Mb) | 9.6 | 9.2 | 5.1 | 5.1 | 3.6 | 1.1 |
| N70 (Mb) | 7.3 | 6.9 | 3.5 | 3.7 | 2.9 | 0.9 |
| N80 (Mb) | 5.2 | 4.4 | 2.0 | 2.7 | 2.1 | 0.6 |
| N90 (Mb) | 3.3 | 2.7 | 2.0 | 1.4 | 1.1 | 0.4 |
| L50 (#) | 60 | 70 | 86 | 120 | 158 | 474 |
| L60 (#) | 82 | 93 | 117 | 161 | 215 | 647 |
| L70 (#) | 108 | 121 | 155 | 214 | 285 | 866 |
| L80 (#) | 146 | 163 | 208 | 285 | 376 | 1,155 |
| L90 (#) | 202 | 227 | 287 | 399 | 522 | 1,612 |

**Supplemental Table 2. Effect of sequencing coverage on assembly contiguity.** To determine a theoretical optimal coverage, we created genome assemblies from subsets of reads. As sequence redundancy decreases, contiguity follows the trend and the number of contigs increases.


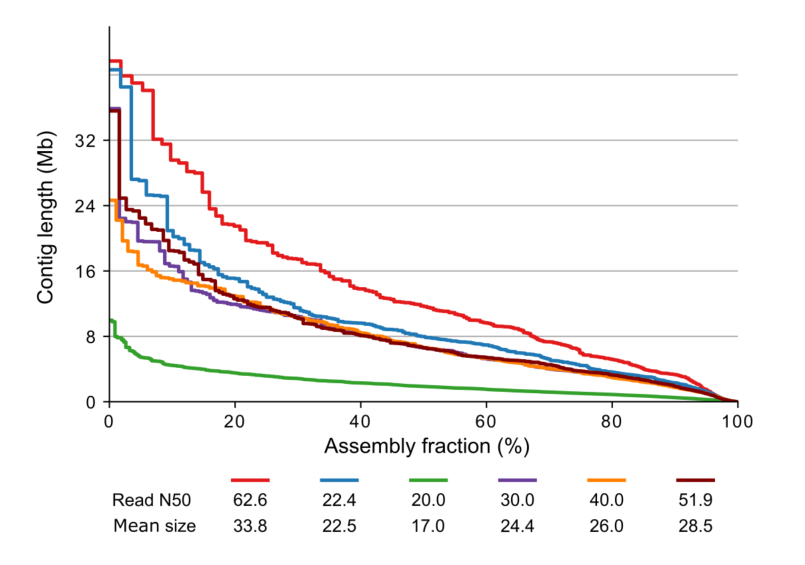


**Supplemental Figure 1. Effect of read length on assembly contiguity.** The plot compares assembly contiguity as a function of the total assembly span. Given the same amount of input bases, assemblies produced with longer reads (read N50 and mean read size are reported below the plot) result in longer contigs.


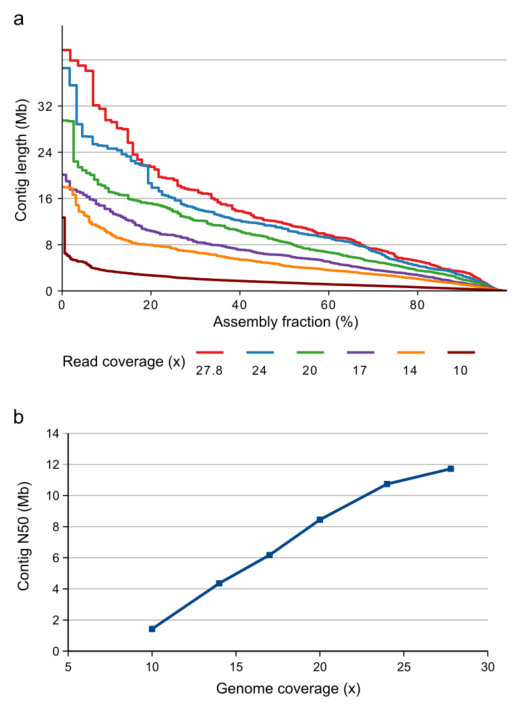


**Supplemental Figure 2. Effect of read coverage on assembly contiguity.** a) The plot compares decreasing contig length (and thus contiguity) as a function of the total assembly span. Given the same mean/N50 read length, assemblies produced with higher coverage are composed by longer and less contigs, resulting in higher contiguity statistics. b) The depletion curve inferred from assemblies at lower coverage hints that higher N50 values that could still be obtained, should supplementary sequence data have been available for this genotype.
