# Supplementary material for: Ultralong Oxford Nanopore Reads Enable the Development of a Reference-Grade Perennial Ryegrass Genome Assembly
Source: Genome Biol Evol. 2021 Jul 10;13(8):evab159. doi: 10.1093/gbe/evab159 (PMC8358221; doi:10.1093/gbe/evab159)
Supplement: evab159_Supplementary_Data [file evab159_supplementary_data.zip › Suppl_material_210731.docx]

**Supplemental data**

**Supplemental materials and method**s

*Origin of the Kyuss genotype*

The sequenced genotype was obtained from anther culture of the *Lolium perenne* genotype DH 6-47 (DH2015 9906 47). Briefly, the appropriate microspore development stage (Begheyn et al. 2017) was assessed using an optical microscope. Once anthers contained mostly late-uninucleate microspores, spikes were harvested and stored in the dark at 4 °C for one to five days. Anther culture was then performed according to (Begheyn et al. 2017). Following regeneration and rooting *in vitro*, single tillers were transferred to the soil and grown in a growth chamber with 14 h light and 8 h darkness at 24 °C. Once fully established in the soil, about ten plants were grown to fully develop biomass.

The plants were genotyped with simple sequence repeat (SSR) markers (primer sequence is reported below). DNA was extracted from fresh leaf material using the Mag-Bind Plant DS Kit, 4x96 on a Thermo Scientific™ KingFisher™ Flex purification system. Three loci heterozygous in the DH 6-47 parent (FA12H05RT048 and FA35E08RT067 from (Saha et al. 2004), and B1A8 from (Lauvergeat et al. 2005)) were amplified using GoTaq Flexi Polymerase (Promega). PCR reactions were performed with the C1000 Touch thermal Cycler (Biorad) in a total volume of 20 μl and containing 15 ng of template DNA. PCR conditions were the following: 5 min at 94 °C for initial denaturation, a touch-down amplification phase of 12 cycles (30 s at 94 °C, 1 min extension: initially 72 °C, decreasing 1 °C every cycle), followed by a second amplification phase (30 sec at 94 °C, 1 min at 60 °C and 1 min at 72 °C for 30 cycles), followed by a final extension at 72°C for 5 min. Upon 1:20 dilution, fragments were separated by 3730 DNA Analyzer (applied Biosystems). Genotypes were called with Geneious 9 (Biomatters Ltd.) A plant (#39) showing only one allele at all three loci was named Kyuss and selected for all downstream analyses.

*DNA extraction, library preparation, and sequencing*
For ONT sequencing, Kyuss DNA was extracted from fully extended leaves according to (Russo et al. 2021). To achieve higher read lengths, we optimized the ONT Genomic DNA by Ligation (SQK-LSK109, version GDE_9063_v109_revU_14Aug2019) protocol (available upon registration at <https://community.nanoporetech.com/>) as described in Supplementary protocol. By means of a wide bore pipette tip, half of the resulting library was loaded into FLO-PRO002 flow cells. The run was paused after 24 hours for a nuclease flush (EXP-WSH003), bringing several thousand pores back to active sequencing state. The second half of the library was injected and a second run was started as previously described. Overall, five PromethION flow cells were used to generate the long-read data for Kyuss. The flow cells were previously identified by ONT as low performing – explaining the low productivity.

The short-read sequencing library was prepared with the TruSeq DNA Nano Library Prep Kit (Illumina, Inc, California, USA). About 200 ng of DNA (extracted with the Mag-Bind Plant DS Kit, see above) were sonicated with the Covaris using settings specific to the fragment size of 500 bp. The fragmented DNA samples were size selected using Sample Purification Beads, end-repaired and polyadenylated. TruSeq adapters containing the index for multiplexing were ligated to the fragmented DNA samples. Fragments containing TruSeq adapters on both ends were selectively enriched by PCR. The quality and quantity of the enriched libraries were validated using a Tapestation (Agilent, Waldbronn, Germany). The product was a smear with an average fragment size of approximately 700 bp. The libraries were normalized to 10nM in Tris-Cl 10 mM, pH8.5 with 0.1% Tween 20. The Novaseq 6000 (Illumina, Inc, California, USA) was used for cluster generation and sequencing according to standard protocol. The library was sequenced with the paired end 150 bp mode.

*Genome size estimation*
The genome size of Kyuss was estimated using CyStain PI Absolute P kit (Sysmex) on a CyFlow Space (Sysmex). Approximately 0.5 cm^2^ of the leaf tissue were chopped with a razor blade in 0.8 ml of the extraction buffer, together with approximately the same size of a tomato (*Solanum lycopersicum*, haploid genome size: ca. 958 Mb, (Doležel et al. 1992)) leaf as internal standard. After incubating on ice for a few minutes, the solution was filtered with 50 µm mesh and 1.6 ml of staining solution with PI and RNase was added to the sample solution. The size of the Kyuss genome was also estimated *in silico* with estimate_genome_size.pl (v0.05, (Luo et al. 2012)) using the short read data and a k-mer values of 19, 21, and 23. The final value was the average of the three estimations. The peaks of the k-mer distribution were produced with Jellyfish (v2.4.2, (Marçais & Kingsford 2011)) count and histo with -C and the specific -m value.

*Genome assembly and quality assessment*
ONT data was basecalled with Guppy (v4.0.14, https://community.nanoporetech.com), keeping reads of 2 kb or longer and having a minimum q-score of 7. Adapter sequences were removed with Porechop (v0.2.4, (Wick et al. 2017)) and sequences shorter than 2 kb were removed. General statistics of the sequencing data were generated with NanoPlot (v1.28.0, (De Coster et al. 2018)). The genome was assembled with Flye (v2.7.1-b1590, (Kolmogorov et al. 2019)) with parameters --genome-size 2500m --min-overlap 10000 --iterations 2. The Shasta (v.0.6.0, (Shafin et al. 2020)) assembly was performed with the Nanopore-Sep2020.conf file and a minimum read length of 14 kb. The theoretical N50 and coverage values attainable with the current genome and dataset were estimated with the fitted model 2.2735 × 10^7^ – 3.0813 × 10^7^ *e*^-0.037x^ (R^2^ 0.9965).

Contig nt-level accuracy was increased by two rounds of polishing with medaka (v1.1.1, https://nanoporetech.github.io/medaka/) and two with Pilon (v1.23, (Walker et al. 2014)). For the latter, the input short read alignment file was obtained with BWA-MEM (v0.7.17-r1188, (Li & Durbin 2010)) and parsed with SAMtools (v1.9, (Li et al. 2009)) view, sort, and index subcommands. To confirm the haploid nature of the assembly and inspect for the occurrence of collapsed regions, the long reads were aligned back to the assembly with minimap2 (v2.17-r974-dirty, (Li 2018)), with -ax map-ont -c --secondary=no parameters. Short reads were also aligned with minimap2, with -a -N 1 --secondary=no -c settings. Alignments were parsed with SAMtools view, sort, and index subcommands; coverage was computed with Bedtools (v2.29.0, (Quinlan & Hall 2010)) makewindows (-w 10000 -s 5000) and coverage (-mean) subcommands. To estimate the nt-level accuracy, the short-read alignment file was parsed with SAMtools the stats subcommand. The assembly completeness was assessed with the KAT tool (v2.4.2, (Mapleson et al. 2017), with -m 23). The k-mer based completeness was 99.39%, with 99.7% of the assembly k-mers present once in the homozygous peak (multiplicity 53) and 78.1% present twice in the repeat peak. The completeness of gene space in the assembly was assessed with BUSCO (v3.0, (Seppey et al. 2019)) in genome mode, with wheat as the model species for AUGUSTUS. Chloroplast and mitochondrial contigs were identified by aligning the assembly (using minimap2) to GenBank accessions NC_019651.1 and JX999996.1, respectively. Contigs with hits at 98% or more similarity were flagged. Contigs of endosymbiont origin were identified by aligning with BLASTN (BLAST+ suite, v2.9.0+, (Camacho et al. 2009)) the contigs to a collection of *Epichloe* and *Neotyphodium* sequences (42,341 ESTs and 4621 nucleotidic sequences downloaded from NCBI nr on November 22, 2018). Hits aligning at ≥96% similarity across ≥60% of the query length on contigs having a mean coverage below 13× or above 80× were removed from the final assembly.

Contigs were assigned to chromosome pseudomolecules with ALLMAPS (v0.7.7 (Tang et al. 2015)) using a genetic linkage map (Pfeifer et al. 2013) and the collinearity with barley as sets of evidence. To map the *Lolium perenne* genetic markers to Kyuss contigs, ESTs were aligned to the Kyuss assembly with TBLASTX and alignments with less than 90% similarity spanning less than 60% query coverage were removed. Collinearity with barley (Mascher et al. 2017) was established by projecting *L. multiflorum* ‘Rabiosa’ gene models (Copetti et al. 2021) to the Kyuss contigs with gmap (2020-10-14, (Wu & Watanabe 2005), -f 2 -n 1 --gff3-add-separators=0 --min-identity=0.95) and by deriving the protein sequences with gffread (part of Cufflinks v.2.2.1, (Trapnell et al. 2010), -M). The primary transcripts of the two proteomes were aligned with DIAMOND (0.9.29,(Buchfink et al. 2015)) and the collinear blocks were determined with MCXScanX (v2, (Wang et al. 2012), -e 15 -k10). When more than four genes of a contig were aligning to a second barley pseudomolecule, the contig was deemed as chimeric and split in two or more sequences. The breakpoint was narrowed down by integrating Shasta assembly’s mapping information to the Flye contigs. To accommodate for the translocation between chromosome 4 and 5, Kyuss Flye contigs with genes whose barley orthologue was spanning models HORVU5Hr1G092630-HORVU5Hr1G116580 were moved to the top of linkage group 4 in reverse order. The genetic map and the collinearity information were integrated with ALLMAPS giving equal weight to the two input sets of evidence.

*Genome annotation*Repeated sequences and transposable elements were annotated by homology with the repeat libraries of (Copetti et al. 2021) using Repeatmasker (v.4.0.6, (Smit A.F.A. et al.), -qq -norna -no_is -gff -cutoff 250 -gccalc -engine ncbi).

Protein-coding genes were annotated using the EVidenceModeler (v1.1.1, (Haas et al. 2008)). Input tracks consisted of (i) *ab initio* gene prediction by AUGUSTUS (v3.3.2, *ab initio* mode, (Stanke & Waack 2003)) using weights trained for Italian ryegrass (Copetti et al. 2021), (ii) proteome alignments of *Brachypodium distachyon* (v3.0, (International Brachypodium Initiative 2010)), barley (Morex v2, (Monat et al. 2019)), wheat (v2.2, (Consortium (IWGSC) 2014)), Italian ryegrass (Copetti et al. 2021) and perennial ryegrass (Blanco‐Pastor et al.) generated using GenomeThreader (v1.7.1, (Gremme et al. 2005)), and (iii) a comprehensive transcriptome set built by PASA (v2.4.1 (Haas et al. 2008)) using genome-guided (Cufflinks v2.2.1, (Trapnell et al. 2010)) and  *de novo* (Trinity v2.1.1 (Haas et al. 2013)) assembled transcripts. To cover as many different organs and experimental conditions as possible, publicly available RNA-Seq experiments were downloaded from SRA (SRP219951, SRP108040, SRP102678, SRP062084 and SRP059405). To retain high confidence gene models, the output of the EVidenceModeler was filtered keeping genes with a cumulative TPM expression level >1 (calculated using Stringtie v2.1.4 (Pertea et al. 2016)) and the selected RNA-Seq experiments, and of which the coding sequence had less than 70% overlap with the annotated repeat regions (see above). Models encoding a TE protein (40% or higher BLASTP similarity to a set of TE coding regions over at least 33 amino acids) were also removed. The resulting 38,868 gene models were further screened for completeness of the gene annotation using BUSCO v3 (Viridiplantae Odb9, (Seppey et al. 2019)) and coreGFs using the PLAZA 2.5 monocots core gene families (Van Bel et al. 2012). More than 87% of the single-copy orthologs were identified in the annotation, and the vast majority (84.2%) were found in a single copy. Fragmented and missing models accounted for 6.5% and 5.9%, respectively. Only 411 out of 7076 gene families were not found, resulting in a 92.2% PLAZA score.

*SSR primers used for genotyping*

FA18-FAM
F: CGAGGTCTCAATCCTCCATT, modified with FAM fluorescent dye for detection on a capillary sequencer
R: GTTTCTTGACAGAGACGACGACGACAT
SSR Motiv: (TCT)6 Reference: FA12H05RT048 (Saha et al., 2004)

FASSR28-HEX
F: TCCTGAGAGACATCGAGCAG, modified with HEX fluorescent dye for detection on a capillary sequencer
R: GTTTCTTTCAAAAGCCCAAACACTTCC
SSR Motiv: (CTGAT)4 Reference: FA35E08RT067 (Saha et al., 2004)

LPSSR55-FAM
F: GACTTTCAGGCATCGGTCAT, modified with FAM fluorescent dye for detection on a capillary sequencer
R: CCCAGCTCCATTCTTAATGC
SSR Motiv: (TG)7 B1A8 (Lauvergeat et al., 2005)

**Supplemental protocol**

**Optimized Oxford Nanopore protocol for sequencing long DNA fragments from grass species**

Based on SQK-LSK109, version GDE_9063_v109_revU_14Aug2019. Modifications are in **bold**.

Original Oxford Nanopore protocol can be found on the website https://community.nanoporetech.com/ (Community login required or registration to login) or available on request – please contact Daniel Frei at Agroscope Switzerland.

1. **HMW DNA enrichment**

- Remove short DNA fragments using the Circulomics Short Read Eliminator Kit (SKU SS-100-101-01, Circulomics Ltd, Baltimore, USA). Follow the supplier’s protocol, leaving the samples in elution buffer at room temperature **overnight**.

1. **Prepare the DNA in nuclease-free water**

- For R9.4.1 flow cells, transfer 1.5 - 2 μg genomic DNA into a 1.5 ml Eppendorf DNA LoBind tube.
- Adjust the volume to 49 μl with Qiagen EB buffer.
- Mix thoroughly by gently tapping the tube to avoid unwanted shearing.
- Spin down briefly in a microfuge.
- In a 0.2 ml thin-walled PCR tube, mix the following:

48 μl DNA
3.5 μl NEBNext FFPE DNA Repair Buffer
2 μl NEBNext FFPE DNA Repair Mix
3.5 μl Ultra II End-prep reaction buffer
3 μl Ultra II End-prep enzyme mix.

- Mix gently by **tapping the tube**, and spin down.
- Using a thermal cycler, incubate at 20°C for **30 minutes** and 65°C for **30 mins**.

1. **AMPure XP bead clean-up**

- Resuspend the AMPure XP beads by vortexing.
- Transfer the DNA sample to a clean 1.5 ml Eppendorf DNA LoBind tube.
- Add 60 μl of resuspended AMPure XP beads to the end-prep reaction and mix by **tapping the tube**.
- Incubate on a Hula mixer (rotator mixer) for **at least** **4 hours at RT**.
- Prepare 500 μl of fresh **65% ethanol** in Nuclease-free water.
- Spin down the sample and pellet on a magnet until eluate is clear and colorless. Keep the tube on the magnet, and pipette off the supernatant.
- Keep the tube on the magnet and wash the beads with 200 μl of freshly prepared **65% ethanol** without disturbing the pellet. Remove the ethanol using a pipette and discard.
- Repeat the previous step.
- Spin down and place the tube back on the magnet. Pipette off any residual ethanol. Allow to dry for ~30 seconds, but do not dry the pellet to the point of cracking.
- Remove the tube from the magnetic rack and resuspend the pellet in 61 μl **Qiagen EB buffer. Incubate for 15 minutes at 55°C then at RT overnight.**
- Pellet the beads on a magnet until the eluate is clear and colorless.
- Remove and retain 61 μl of eluate into a clean 1.5 ml Eppendorf DNA LoBind tube.
- Quantify 1 μl of eluted sample using a Qubit fluorometer (1.4 – 2 μg is targeted to split the library later on).

1. **Adapter ligation and clean-up**

- Although the recommended 3rd party ligase is supplied with its own buffer, the ligation efficiency of Adapter Mix (AMX) is higher when using Ligation Buffer supplied within the Ligation Sequencing Kit.
- Spin down the Adapter Mix (AMX) and Quick T4 Ligase, and place on ice.
- Thaw Ligation Buffer (LNB) at RT, spin down and mix by pipetting. Due to viscosity, vortexing this buffer is ineffective. Place on ice immediately after thawing and mixing.
- Thaw the Elution Buffer (EB) at RT, mix by vortexing, spin down and place on ice.
- To enrich for DNA fragments of 3 kb or longer, thaw one tube of Long Fragment Buffer (LFB) at RT, mix by vortexing, spin down and place on ice.
- In a 1.5 ml Eppendorf DNA LoBind tube, mix in the following order:

60 μl DNA sample from the previous step
25 μl Ligation Buffer (LNB)
10 μl NEBNext Quick T4 DNA Ligase
5 μl Adapter Mix (AMX).

- Mix gently by **tapping the tube**, and spin down.
- Incubate the reaction for **60 minutes** at RT.
- Resuspend the AMPure XP beads by vortexing.
- Add 40 μl of resuspended AMPure XP beads to the reaction and mix by **tapping the tube**.
- Incubate on a Hula mixer (rotator mixer) for **at least 4 hours** at RT.
- Spin down the sample and pellet on a magnet. Keep the tube on the magnet, and pipette off the supernatant.
- Wash the beads by adding 250 μl Long Fragment Buffer (LFB). Resuspend the beads by **gently tapping** the tube. It could take several minutes until resuspension is complete.
- Once completely resuspended, spin down, then return the tube to the magnetic rack and allow the beads to pellet. Remove the supernatant using a pipette and discard.
- Repeat the previous step.
- Spin down and place the tube back on the magnet. Pipette off any residual supernatant. Allow to dry for ~30 seconds, do not dry the pellet to the point of cracking.
- Remove the tube from the magnetic rack and resuspend pellet in 25 μl Elution Buffer (EB). **Incubate for 15 minutes at 55°C, then at RT overnight.**
- Pellet the beads on a magnet until the eluate is clear and colorless.
- Remove and retain 25 μl of eluate containing the DNA library into a clean 1.5 ml Eppendorf DNA LoBind tube.
- Quantify 1 μl of eluted sample using a Qubit fluorometer (1.2-1.6 μg).

1. **Priming and loading the flow cell**

- Thaw the Sequencing Buffer (SQB), Loading Beads (LB), Flush Tether (FLT) and one tube of Flush Buffer (FB) at RT.
- Mix the Sequencing Buffer (SQB), Flush Buffer (FB) and Flush Tether (FLT) tubes by vortexing and spin down at RT.
- To prepare the flow cell priming mix, add 30 μl of thawed and mixed Flush Tether (FLT) directly to 1 tube of thawed and mixed Flush Buffer (FB), and mix by vortexing.
- Load the flow cell(s) into the docking ports within the PromethION.
- Prime the flow cell using the following steps, taking care to avoid the introduction of air bubbles.
- Turn the valve to expose the inlet port. A small tract of air will be visible beyond the inlet port. Draw back a small volume to remove any air bubbles (a few μls): Set a P1000 pipette to 200 μl, insert the tip into the inlet port, turn the wheel until the dial shows 220-230 μl, or until you can see a small volume of buffer entering the pipette tip.
- Using a P1000 pipette, flush 500 μl of the Priming Mix into the inlet port of the flow cell, avoiding the introduction of air bubbles.
- Wait five minutes. During this time, you can prepare your library for loading as described in the next steps.
- Repeat the priming step with another 500 μl Priming Mix flush.
- Thoroughly mix the contents of the Sequencing Buffer (SQB) and Loading Beads (LB) tubes by vortexing.
- In a new tube, prepare the library for loading as follows:

75 μl SQB
51 μl LB
24 μl DNA library **(600-800ng dilute library 1:1 with ONT EB Buffer).**

- Load the 150 μl of your diluted library through the inlet port. **Load your sample with a wide bore pipette tip**.
- Close the valve to seal the inlet port and close the PromethION lid when ready.
- Wait a **minimum of 30 minutes** after loading the flow cells onto the PromethION before starting the run. This will help to increase the sequencing output.
- After 24 h, pause the run.

1. **Flushing a PromethION flow cell**

Preparation to run the washing procedure. (EXP-WSH003)

- Place the tube of Wash Solution A on ice. Do not vortex the tube.
- Thaw one tube of Wash Solution B at RT.
- Mix the contents of Wash Solution B thoroughly by vortexing, spin down briefly and place on ice.
- In a clean 1.5 ml Eppendorf DNA LoBind tube, prepare the following Wash Mix:

20 μl Wash Solution A (A)
380 μl Wash Solution B (B).

- Mix well by pipetting, and place on ice. Do not vortex the tube.
- Pause the sequencing run in MinKNOW and leave the flow cell in the device.
- Ensure the inlet port is closed and remove any buffer from the waste port at the top of the flow cell.
- Rotate the inlet port cover clockwise to reveal the inlet port. A small tract of air may be visible beyond the inlet port. If necessary, using a P1000 draw back a small volume to remove any air (a few µl). Set a P1000 pipette to 200 µl, insert the tip into the inlet port, turn the wheel until the dial shows 220-230 µl, or until you can see a small volume of buffer/liquid entering the pipette tip.
- Open the inlet port and load 400 µl of the prepared Wash Mix into the flow cell via the inlet port, avoiding the introduction of air.
- Close the inlet port and wait for 30 minutes.
- Inject other half of the library (600-800 ng) according to priming and loading the flow cell.
- Resume sequencing as above.

**Supplemental reference**s

Begheyn RF, Roulund N, Vangsgaard K, Kopecký D, Studer B. 2017. Inheritance patterns of the response to in vitro doubled haploid induction in perennial ryegrass (Lolium perenne L.). Plant Cell Tiss Organ Cult. 130:667–679. doi: 10.1007/s11240-017-1255-y.

Blanco‐Pastor JL et al. Canonical correlations reveal adaptive loci and phenotypic responses to climate in perennial ryegrass. Molecular Ecology Resources. n/a. doi: https://doi.org/10.1111/1755-0998.13289.

Buchfink B, Xie C, Huson DH. 2015. Fast and sensitive protein alignment using DIAMOND. Nature Methods. 12:59–60. doi: 10.1038/nmeth.3176.

Camacho C et al. 2009. BLAST+: architecture and applications. BMC Bioinformatics. 10:421. doi: 10.1186/1471-2105-10-421.

Consortium (IWGSC) TIWGS. 2014. A chromosome-based draft sequence of the hexaploid bread wheat (Triticum aestivum) genome. Science. 345:1251788. doi: 10.1126/science.1251788.

Copetti D et al. 2021. Evidence for high intergenic sequence variation in heterozygous Italian ryegrass (Lolium multiflorum Lam.) genome revealed by a high-quality draft diploid genome assembly. bioRxiv. 2021.05.05.442707. doi: 10.1101/2021.05.05.442707.

De Coster W, D’Hert S, Schultz DT, Cruts M, Van Broeckhoven C. 2018. NanoPack: visualizing and processing long-read sequencing data. Bioinformatics. 34:2666–2669. doi: 10.1093/bioinformatics/bty149.

Doležel J, Sgorbati S, Lucretti S. 1992. Comparison of three DNA fluorochromes for flow cytometric estimation of nuclear DNA content in plants. Physiologia Plantarum. 85:625–631. doi: https://doi.org/10.1111/j.1399-3054.1992.tb04764.x.

Gremme G, Brendel V, Sparks ME, Kurtz S. 2005. Engineering a software tool for gene structure prediction in higher organisms. Information and Software Technology. 47:965–978. doi: 10.1016/j.infsof.2005.09.005.

Haas BJ et al. 2008. Automated eukaryotic gene structure annotation using EVidenceModeler and the Program to Assemble Spliced Alignments. Genome Biol. 9:R7. doi: 10.1186/gb-2008-9-1-r7.

Haas BJ et al. 2013. De novo transcript sequence reconstruction from RNA-Seq: reference generation and analysis with Trinity. Nat Protoc. 8. doi: 10.1038/nprot.2013.084.

International Brachypodium Initiative. 2010. Genome sequencing and analysis of the model grass Brachypodium distachyon. Nature. 463:763–768. doi: 10.1038/nature08747.

Kolmogorov M, Yuan J, Lin Y, Pevzner PA. 2019. Assembly of long, error-prone reads using repeat graphs. Nature Biotechnology. 37:540–546. doi: 10.1038/s41587-019-0072-8.

Lauvergeat V, Barre P, Bonnet M, Ghesquière M. 2005. Sixty simple sequence repeat markers for use in the Festuca–Lolium complex of grasses. Molecular Ecology Notes. 5:401–405. doi: https://doi.org/10.1111/j.1471-8286.2005.00941.x.

Li H. 2018. Minimap2: pairwise alignment for nucleotide sequences. Bioinformatics. 34:3094–3100. doi: 10.1093/bioinformatics/bty191.

Li H et al. 2009. The Sequence Alignment/Map format and SAMtools. Bioinformatics. 25:2078–2079. doi: 10.1093/bioinformatics/btp352.

Li H, Durbin R. 2010. Fast and accurate long-read alignment with Burrows-Wheeler transform. Bioinformatics. 26:589–595. doi: 10.1093/bioinformatics/btp698.

Luo R et al. 2012. SOAPdenovo2: an empirically improved memory-efficient short-read de novo assembler. GigaScience. 1:18. doi: 10.1186/2047-217X-1-18.

Mapleson D et al. 2017. KAT: a K-mer analysis toolkit to quality control NGS datasets and genome assemblies. Bioinformatics. 33:574–576. doi: 10.1093/bioinformatics/btw663.

Marçais G, Kingsford C. 2011. A fast, lock-free approach for efficient parallel counting of occurrences of k-mers. Bioinformatics. 27:764–770. doi: 10.1093/bioinformatics/btr011.

Mascher M et al. 2017. A chromosome conformation capture ordered sequence of the barley genome. Nature. 544:427–433. doi: 10.1038/nature22043.

Monat C et al. 2019. TRITEX: chromosome-scale sequence assembly of Triticeae genomes with open-source tools. Genome Biology. 20:284. doi: 10.1186/s13059-019-1899-5.

Pertea M, Kim D, Pertea GM, Leek JT, Salzberg SL. 2016. Transcript-level expression analysis of RNA-seq experiments with HISAT, StringTie and Ballgown. Nature Protocols. 11:1650–1667. doi: 10.1038/nprot.2016.095.

Pfeifer M et al. 2013. The perennial ryegrass GenomeZipper: targeted use of genome resources for comparative grass genomics. Plant Physiol. 161:571–582. doi: 10.1104/pp.112.207282.

Quinlan AR, Hall IM. 2010. BEDTools: a flexible suite of utilities for comparing genomic features. Bioinformatics. 26:841–842. doi: 10.1093/bioinformatics/btq033.

Russo A, Potente, Giacomo, Mayionade, Baptiste. 2021. HMW DNA extraction from diverse plants species for PacBio and Nanopore sequencing. doi: 10.17504/protocols.io.5t7g6rn.

Saha MC et al. 2004. Tall fescue EST-SSR markers with transferability across several grass species. Theor Appl Genet. 109:783–791. doi: 10.1007/s00122-004-1681-1.

Seppey M, Manni M, Zdobnov EM. 2019. BUSCO: Assessing Genome Assembly and Annotation Completeness. In: Gene Prediction: Methods and Protocols. Kollmar, M, editor. Methods in Molecular Biology Springer: New York, NY pp. 227–245. doi: 10.1007/978-1-4939-9173-0_14.

Shafin K et al. 2020. Nanopore sequencing and the Shasta toolkit enable efficient de novo assembly of eleven human genomes. Nature Biotechnology. 38:1044–1053. doi: 10.1038/s41587-020-0503-6.

Smit A.F.A., Hubley R., Green P. RepeatMasker. http://repeatmasker.org.

Stanke M, Waack S. 2003. Gene prediction with a hidden Markov model and a new intron submodel. Bioinformatics. 19:ii215–ii225. doi: 10.1093/bioinformatics/btg1080.

Tang H et al. 2015. ALLMAPS: robust scaffold ordering based on multiple maps. Genome Biology. 16:3. doi: 10.1186/s13059-014-0573-1.

Trapnell C et al. 2010. Transcript assembly and quantification by RNA-Seq reveals unannotated transcripts and isoform switching during cell differentiation. Nature Biotechnology. 28:511–515. doi: 10.1038/nbt.1621.

Van Bel M et al. 2012. Dissecting Plant Genomes with the PLAZA Comparative Genomics Platform. Plant Physiology. 158:590–600. doi: 10.1104/pp.111.189514.

Walker BJ et al. 2014. Pilon: An Integrated Tool for Comprehensive Microbial Variant Detection and Genome Assembly Improvement. PLOS ONE. 9:e112963. doi: 10.1371/journal.pone.0112963.

Wang Y et al. 2012. MCScanX: a toolkit for detection and evolutionary analysis of gene synteny and collinearity. Nucleic Acids Res. 40:e49. doi: 10.1093/nar/gkr1293.

Wick RR, Judd LM, Gorrie CL, Holt KE. 2017. Completing bacterial genome assemblies with multiplex MinION sequencing. Microbial Genomics,. 3:e000132. doi: 10.1099/mgen.0.000132.

Wu TD, Watanabe CK. 2005. GMAP: a genomic mapping and alignment program for mRNA and EST sequences. Bioinformatics. 21:1859–1875. doi: 10.1093/bioinformatics/bti310.
